# CogCARE: Tailoring Cognitive Rehabilitation For Black/African American Older Adults

**DOI:** 10.1093/geroni/igaf122.4362

**Published:** 2025-12-31

**Authors:** Dumichel Harley, Yoojee Kim, Ryan Mace

**Affiliations:** Massachusettes General Hospital BHX-1, Cambridge, Massachusetts, United States; Massachusetts General Hospital, Boston, Massachusetts, United States; Massachusetts General Hospital, Boston, Massachusetts, United States

## Abstract

Cognitive rehabilitation (CR) programs provide cognitive strategies for individuals with cognitive decline and improve cognitive/functional outcomes. African American (AA) individuals are disproportionately affected by dementia and have cultural preferences not addressed in CR programs currently. Resources are needed to aid this underserved population. This concurrent mixed methods study guided development of CogCare, which would provide tailored CR strategies to improve stress/mood and cognitive/daily functioning for older AA adults with mild/moderate dementia and their caregivers. Older adults and dementia caregivers (N = 16 older adults; N = 3 caregivers) first completed self-report measures of emotional/cognitive/daily functioning, and were administered a cognitive screener (MCQ, CRS, PROMIS-CF). They then participated in one-time focus groups (N = 5). Select members participated in one-on-one interviews to understand their experiences with dementia/dementia caregiving. Qualitative analyses identified needs/knowledge/cultural preferences of this population. Quantitative analysis explored correlations between treatment targets. We triangulated the mixed methods results to ensure that tailoring of CogCare is empirically/culturally supported. Thematic analyses highlighted the following themes: (1) CR strategies, (2) cultural preferences, (3) caregiver considerations, (4) treatment barriers, and (5) awareness/recognition of cognitive decline. Quantitative analyses showed that (a) greater compensation strategy use correlated with better self-perceptions of cognitive functioning (p < 0.001) and (b) caregiver burden and competence correlated with a loss of sense of self and personal growth, respectively (p = .004; p = .000). Participant feedback underscores the demand for accessible, community-informed CR. These findings will inform an open pilot to evaluate feasibility and refine CogCare prior to conducting a randomized trial.

